# Impact of the COVID-19 pandemic on stress and emotional reactions in Israel: a mixed-methods study

**DOI:** 10.1093/inthealth/ihaa081

**Published:** 2020-10-13

**Authors:** Inbar Levkovich, Shiri Shinan-Altman

**Affiliations:** Faculty of Graduate Studies, Oranim Academic College of Education, Kiryat Tivon 36006, Israel; The Louis and Gabi Weisfeld School of Social Work Bar Ilan University, Max and Anna Web st., Ramat Gan 5290002, Israel

**Keywords:** COVID-19, emotional reactions, perceived susceptibility, Israel, mixed-methods

## Abstract

**Background:**

The COVID-19 pandemic has had a profound impact worldwide. This study sought to assess the pandemic's psychological impact on the Israeli public.

**Methods:**

Using mixed methods we assessed Israeli adults during the COVID-19 outbreak. In the quantitative study, participants (N=1407) completed an online battery of measures assessing psychological variables and perceived threat related to COVID-19. Statistical analyses included tests for between-group differences and Pearson correlations. The qualitative study entailed in-depth, semistructured interviews conducted by telephone (N=38).

**Results:**

The quantitative findings indicate that about 48% of the public had negative emotional reactions and 20% perceived they were liable to contract the virus. Moreover, a positive correlation was found between these feelings and the degree of perceived threat. Three major themes emerged from the qualitative study: 1) a sense of shock and chaos; 2) gradual adjustment to the new reality; and 3) fears and concerns for self and family members. The study's results revealed the following sources of participants’ emotional responses and sense of threat: health concerns regarding themselves and their loved ones; employment concerns; problems with children and spouses caused by being together at home; and difficulties entailed in working at home.

**Conclusions:**

The study reveals many of the psychological variables and perceived threats related to COVID-19 in Israel. While social distancing may make people feel safer, it can also increase their feelings of isolation, stress and frustration and cause difficulties in many life situations. The findings point to the necessity of addressing the public's perceived susceptibility and emotional reactions about COVID-19.

## Introduction

The 2019 coronavirus disease (COVID-19) caused by the novel coronavirus (SARS-CoV-2) began in the city of Wuhan in China and spread quickly around the world, generating a global health crisis of massive proportions. As a result of this pandemic, people found themselves forced to cope with new emotional challenges and particularly with feelings of stress, uncertainty and fear. COVID-19 poses a real threat to physical and emotional health.^1^ Indeed, previous research on viruses shows that pandemic situations exert an emotional impact on people's levels of stress and resilience.^2^ People's fears that they themselves or those close to them will become ill or will die may generate psychological effects. Feelings of fear and helplessness together with reports of shortages in medical supplies are liable to increase stress.^3,4^ For example, during a flu outbreak about 10–30% of the general public reported major fears of contracting the disease.^5^ When schools and shops were closed during the SARS outbreak, people reported an increase in negative emotions and the level of psychiatric morbidity also increased.^6^ Women, older adults, educated individuals, anxious individuals and those who had symptoms of the virus complied more strictly with precautionary measures against the virus.^7,8^ Moreover, research that examined emotional responses to the Ebola virus revealed cases of depression, anxiety, difficulties in concentration and a sense of grief.^9^

A study of COVID-19 conducted in China found that among 1210 respondents from the general public, 53.8% rated the psychological impact of the outbreak as moderate or severe, 16.5% reported moderate to severe depressive symptoms, 28.8% reported moderate to severe anxiety symptoms and 8.1% reported moderate to severe stress levels.^10^ Another study that examined 52 730 participants in Hong Kong found that 35% reported feeling stressed about COVID-19, with women reporting higher levels of stress than men.^1^

Israel's population is 9.1 million (median age: 30 y).^11^ The first case of COVID-19 in Israel was diagnosed towards the end of February 2020. By the end of the research period, thousands were in isolation at home, 13 930 were diagnosed with the virus and, according to Ministry of Health figures for 21 April 2020, 181 had died from the disease.^12^

Like other countries, Israel implemented diverse containment measures, including quarantines and closures.^13^ In mid-March, the government decided to close down the education system and to prohibit gatherings of >10 people. Entertainment and other public venues were closed, including restaurants, movie theatres, gyms, shopping centres, places of worship, beaches and parks. People were advised to avoid large gatherings at work and to maintain a distance of at least 2 m between employees.^12^

The Israeli Ministry of Health regularly released and updated guidelines and instructions to explain the new daily routine to the general public (e.g. taking precautions such as frequently washing hands with soap and water or alcohol-based hand sanitisers, avoiding close contact with people showing symptoms, refraining from shaking hands and covering the mouth and nose when coughing or sneezing).^12^ The ongoing rise in the numbers of suspected and diagnosed cases is also liable to affect the public's estimations of the severity and controllability of the virus.^14–17^ In an Israeli study conducted among 639 participants, gender, sociodemographic status, chronic illness, being in an at-risk group and having a family member who had died of COVID-19 were positively associated with fear of COVID-19, and fear levels were associated with anxiety, stress and depression.^18^ Therefore, the current study used quantitative methods to examine the Israeli public's emotional reactions and perceived susceptibility to COVID-19. It then employed qualitative methods to investigate how the Israeli public perceived their experiences with the virus, how they expressed their emotions on this matter and how they perceived their own coping methods.

## Methods

### Procedure and participants

The quantitative study entailed a cross-sectional online survey conducted among 1407 participants in Israel between 12 and 21 March 2020 (Table 1). To minimise personal contact during the outbreak, the questionnaires were administered online through the Qualtrics online platform (www.qualtrics.com). A link to the electronic survey was distributed via Facebook or WhatsApp. Before completing the survey, participants were asked to read an informed consent form and to indicate that they agreed to participate in the study. Only after giving their consent were they permitted to answer the questionnaire. To be included in the study participants had to be aged ≥18 y and able to speak Hebrew. Exclusion criteria were: being aged <18 y, which is the cutoff age for requiring parental consent; giving responses that conformed to a similar pattern (e.g. choosing the same answer across many consecutive items or for the entire questionnaire); or failing to complete the entire questionnaire (specifically, people who began filling out the questionnaire but did not complete it did not participate in the study). Participants who completed the entire questionnaire but omitted a single demographic item, such as gender or health status, did participate in the study.

**Table 1.** Characteristics of participants in the quantitative study (N=1407)

Characteristic	N (%)
Gender (%)	
Male	282 (20.1)
Female	1119 (79.9)
Mean age years (SD), range	40.97 (14.66), 18–97
Mean number of years of education (SD), range	16.44 (3.66), 9–30
Marital status (%)	
Married	879 (62.7)
Divorced	81 (5.8)
Widowed	24 (1.7)
Single	380 (27.1)
Other	37 (2.6)
Mean number of children (SD), range	2.18 (1.39), 0–9
Health problems (%)	
Yes	214 (15.3)
No	1186 (84.7)
Health status (%)	
Poor	19 (1.4)
Fair	276 (19.6)
Good	1108 (79.0)
Resources that can make it easier to cope with COVID-19 (%)	
More information regarding COVID-19	260 (19.4)
Professional support	172 (12.8)
Layman support	143 (10.7)
Working from home	529 (39.4)
Other	237 (17.7)

The qualitative study examined a sample from the Israeli public (N=38; 29 women and 9 men). Participants were contacted via adverts on social media asking them to take part in a research study and a personal telephone interview. Those who were interested sent an email to the researcher, who contacted them and explained the study to them. A telephone interview was set up with those who agreed to participate. Before the interview, the consent form was read aloud to the participants, who then gave their informed consent. The interviews were conducted by telephone due to the closure and the guidelines regarding social distancing. Table 2 provides details regarding the background characteristics of the sample. Participants ranged in age from 20 to 53 y (median [M]=33.26, SD=10.15) and had zero to four children (M=1.29, SD=1.4); 47.4% were married, 47.3% were single and 5.3% were divorced.

**Table 2.** Demographic characteristics of participants in the qualitative study (N=38)

Characteristic	N	%
Age (mean and standard	M=33.26, SD=10.15	
deviation; years)		
Number of children (mean	M=1.29, SD=1.4	
and standard deviation)		
Gender		
Men	9	23.7%
Women	29	76.3%
Marital status		
Married	18	47.3%
Single	18	47.3%
Divorced	2	5.3%
Occupation		
Student	11	28.9%
Salaried	25	65.8%
Self-employed	2	5.3%

## Phase 1: Quantitative study

### Measures

Perceived susceptibility was assessed based on previous studies conducted among the general public (e.g.^19^) using a one-item measure to examine participants’ perceived likeliness of contracting the virus (e.g. ‘In your opinion, how likely is it that you will contract COVID-19?’).^17^ Participants answered on a five-point Likert-type scale ranging from 1=not at all likely to 5=very likely.

Emotional reactions to COVID-19 were assessed based on previous studies conducted among the general public (e.g.^19^) using three questions related to worry, fear and stress caused by COVID-19 (e.g. ‘To what extent do you worry about COVID-19?’).^17^ Participants answered on a five-point Likert-type scale, ranging from 1=not at all to 5=very much. A composite index of the averages of all items was created, with a higher score indicating higher levels of negative emotional reactions toward COVID-19. The internal consistency of the index was excellent (Cronbach's α=0.94).

Sociodemographic variables included gender, age, years of education, marital status (married/divorced/widowed/single/other), number of children, medical problems (yes/no), health status (poor/fair/good), home isolation since the COVID-19 outbreak (yes/no), resources that would make coping with COVID-19 easier (more information regarding COVID-19/professional support/lay support/working from home/other).

### Statistical analyses

The data were analysed using SPSS version 25 (IBM, Armonk, NY, USA). Descriptive statistics were used to describe participants’ demographic characteristics and the research variables. Spearman correlations were calculated to assess the associations between the research variables and the Bonferroni correction for multiple comparisons was applied.

## Phase 2: Qualitative study

### Methods

The study adopted a qualitative-phenomenological approach.^20^ This type of approach attempts to obtain an in-depth understanding of the studied phenomenon by entering the world and experiences of the participants. Such a paradigm facilitates examining the voices and experiences of the informants as they choose to express them, thus providing a deeper understanding of the interviewees and arriving at insights that give meaning to multidimensional phenomena.^21^

### Procedure and instrument

The research instrument was a semistructured, in-depth questionnaire. The interviewer encouraged participants to talk about their experiences in their own words. The interviews were conducted based on an interview guide (see Appendix 1) that included significant key areas, yet was flexible enough to allow both the development of a dialogue between interviewer and interviewee and for meaningful self-expression.^22^ All interviews were conducted by telephone, audio-recorded and subsequently transcribed. Participants gave their consent to record the interviews. Each interview lasted for 30 min. Data collection and analysis proceeded until theoretical saturation was reached (i.e. additional interviews yielded no new material for analysis).

### Data analysis

The content analysis in this study included the following stages: 1) open coding: the principal investigator first read each interview transcript line by line, jotting down notes to capture and identify initial units of meaning (categories) emerging from the data; 2) the same researcher reviewed the major themes and discussed them with the other researcher; 3) axial coding: upon reading the transcripts a second time, the researchers gradually detected associations between themes and subthemes related to context and content. They compared all completed interviews to consolidate meanings and arrive at a theoretical construct; and 4) integration: the core themes or main categories emerging from the data were reordered conceptually and placed back into context, making it possible to analyse and integrate large amounts of data and to generate abstractions and interpretations.^22^

## Results

### Quantitative phase results

This study was a cross-sectional online survey conducted among 1407 participants in Israel. The majority of the respondents were female (80%). Their mean age was 41 (18–97) y and they had an average of about 16.5 (9–30) y of education. Most were married (63%) and had an average of two children. About 85% reported having no health problems and about 80% reported their health status as good. Moreover, participants indicated that working from home was their preferred resource in coping with COVID-19.

Table 3 summarises the means, SDs, ranges and Spearman correlations of the study variables. The mean scores for emotional reactions (M=2.72, SD=0.93, range 1–5) and perceived susceptibility were about mid-scale (M=3.25, SD=1.14, range 1–5). A positive association was found between perceived susceptibility and emotional reactions to COVID-19 (r=0.30, p<0.001). Moreover, perceived susceptibility exhibited negative associations with age, gender and health status, indicating that those who were older and female, and who perceived that their personal health status was not good, reported higher perceived susceptibility. Likewise, emotional responses exhibited negative associations with age, gender, marital status and health status, so that participants who were older, female and unmarried, and who perceived that their personal health status was not good, reported higher emotional reactions.

**Table 3.** Spearman correlations, means, SDs and ranges of study variables (n=1407)

Variable	1	2	3	4	5	6	7
1. Age, y	-						
2. Gender	0.19***	-					
3. Marital status	0.40***	0.08	-				
4. Pre-existing conditions (health problems)	0.21***	0.05	-0.02	-			
5. Perceived health status	-0.08**	.01	0.03	-0.42***	-		
6. Perceived susceptibility	-0.10**	-0.10**	-0.02	0.06	-0.16***	-	
7. Emotional reactions	-0.24***	-0.22**	-0.11***	0.07	-0.12***	0.30***	-
Mean	40.97	0.20	0.63	0.15	2.78	2.72	3.25
SD	14.66	0.40	0.48	0.36	0.45	0.93	1.14
Possible range	-	-	-	-	1–3	1–5	1–5
Actual range	18–97	-	-	-	1–3	1–5	1–5

Bonferroni correction for multiple comparisons: **p<0.002, ***p<0.001

Gender: 1-male, 0-female

Marital status: 1-married, 0-not married

Pre-existing conditions (health problems): 1-yes, 0-no

Perceived health status: 3-good, 2-fair, 1-poor

Perceived susceptibility: 5-high, 1-low

Table 4 shows the two multiple hierarchical regressions calculated for perceived susceptibility and emotional reactions. Background and health-related variables were entered in the first step, and perceived susceptibility and negative emotional reactions were added in the second step. The results show that age and perceived health status are negatively related to perceived susceptibility, such that perceived susceptibility is higher for women and for participants whose perceived health status is lower. Age, gender and perceived susceptibility are related to emotional reactions, such that emotional reactions are higher among younger participants, women and participants with higher perceived susceptibility.

**Table 4.** Multiple hierarchical regressions for perceived susceptibility and emotional reactions (n=1407)

	Perceived susceptibility			Emotional reactions		
	B	SE	β	B	SE	β
Step 1						
Age, y	-0.01	0.01	-0.11***	-0.01	0.01	-0.19***
Gender	-0.16	0.07	-0.07	-0.45	0.08	-0.16***
Marital status	0.08	0.06	0.04	-0.05	0.07	-0.02
Pre-existing conditions (health problems)	0.03	0.09	0.01	0.29	0.10	0.09
Perceived health status	-0.39	0.07	-0.19***	-0.16	0.08	-0.06
Step 2						
Perceived susceptibility	–	–	–	0.33	0.03	0.27***
Adj. *R*^2^	0.05, p<0.001			0.18, p<0.001		

***p<0.001

Gender: 1-male, 0-female

Marital status: 1-married, 0-not married

Pre-existing conditions (health problems): 1-yes, 0-no

Perceived health status: 3-good, 2-fair, 1-poor

Perceived susceptibility: 5-high, 1-low

**Table 5.** Classification of main categories and subcategories

Main categories	Subcategories
Sense of shock and chaos	• Information search• Sense of shock, confusion, frustration• Sense of lack of control and helplessness regarding the need for behavioural changes
Gradual adjustment to the new reality	• Internalising the guidelines and changing one's customary lifestyle• Changes in the family—the children are at home• Changes in the couple relationship—the members of the couple are together all the time; one or both may be unemployed
Fears and concerns for self and family	• Concerns for own health and health of those close to them• Many described concerns for older relatives or those with illnesses in high-risk groups• Concerns affect behaviour: sleep disruptions, restlessness, irritability, difficulty performing tasks• Depression, anxiety, sadness, loneliness alongside anxiety, uncertainty and helplessness

### Qualitative phase results

The findings of the qualitative study yielded three main themes: 1) a sense of shock and chaos; 2) gradual adjustment to the new reality; and 3) fears and concerns for themselves and their loved ones.

### Theme 1–‘I'm losing control’: sense of shock and chaos

All 38 participants in the qualitative phase described how their lives had changed overnight. This change was drastic, surprising, powerful and difficult to contain. When the COVID-19 epidemic broke out in China, the participants felt the virus was far away and they were immune from it. As the epidemic got closer, reaching European countries and also Israel, the respondents’ sense of shock and serious concern rose. People received their information from diverse media sources. Some of these sources were reliable, such as the Israeli Ministry of Health. Other less reliable sources spoke of conspiracy theories and a feeling that the world was coming to an end. Moreover, the guidelines provided by the Ministry of Health were not always clear to everyone, leading to confusion, information chaos and inner upheaval.

*COVID-19 made its surprising debut on the world stage only a few months ago, a short period that now feels like an eternity. Initially we asked, ‘What is this? How should we respond to this event?’ We were confused, uncertain, aware that this is a major event, something new that we've never experienced. I felt a tremendous need to follow the news and learn what was happening in the world in order to dispel my sense of uncertainty, to understand the repercussions, to learn how this will affect us and understand how to behave* (47-y-old married man, two children).

Twenty-two participants indicated that their feelings ranged from a sense of indifference to denying the situation and continuing as usual. They then began to internalise the difficult situation as it filtered down to them. They were overcome by a sense of lack of control and helplessness that necessitated changes in their behaviour.

*I was overcome by tremendous fear due to COVID-19, fear of contracting the disease, fear of the new reality. I felt helpless and it's hard for me to function in this new reality that has been forced upon me …. I thought the situation would pass quickly and I never thought it would reach such great proportions that the entire country would be shut down* (26-y-old single woman).

Twenty-nine of the participants described being shocked and confused. They searched for a direction to follow and wondered how they should behave and what they should do. The situation generated feelings of frustration and of being stuck.

*Since the COVID-19 outbreak, I have been feeling very frustrated and overwhelmed. The spread of the virus put me in a state of shock, I didn't know how to digest this new reality that until now I've never had to face, how to respond to the situation as it is, how I should act. I didn't know how to divide up my time and what I should do. The virus put everything on hold for me* (53-y-old divorced woman, three children).

### Theme 2–‘Recalculating the route’: gradual adjustment to the new reality

Most of the participants (32) indicated that after their initial feelings of shock, they found themselves gradually adjusting to the new reality that had been forced upon them by COVID-19 and the Ministry of Health guidelines. Adjustment difficulties stemmed from having to remain within their own household, limit their movements and, for some, stop working. The participants described how they internalised these guidelines and made changes to their familiar lifestyle.

*As the situation developed and the virus began to spread, I saw how everything around me began to close and shut down… I began to think differently, to conduct myself differently, to leave the house less frequently, to pay much more attention to maintaining hygiene than usual, to keep my distance from people, to refrain from touching, even my own family. My life routine has changed. I leave the house only if I need something, groceries or the pharmacy or other urgent necessities* (32-y-old married man, one child).

The main issue among all 20 research participants who had children focused on how to maintain a daily routine. Before this happened, the children were at school, attended afterschool activities and met friends. Now parents were charged with the difficult task of mediating this new situation for their children while at the same time maintaining a routine without any clear or defined framework.

*One day, out of the blue, without any advanced warning or chance to prepare, I found myself in a new reality that forced me to recalculate my route, and fast. I found myself at home with my family and I had to be their anchor at a time when everything around us is unknown and uncertain. How can I manage under these circumstances? How can I explain this strange new reality to my children? How can I maintain some sort of sane schedule when everything is so unclear? Yet despite all these complexities, I understood that this is a good time and wonderful opportunity for a mother with a family* (42-y-old married woman, three children).

The parents described how they are adjusting to the new situation by setting up a regular routine that includes chores, arts and crafts, cooking activities, home exercise workouts and cleaning. Most of the children study via distance learning and many of the parents work from home. Most of the parents (16) described being exhausted by these diverse roles and by the physical and emotional burden they entailed.

*I found that the easiest way for us to cope with the isolation was to set up a regular daily routine. When this first began, we tidied up the house, set up an arts and crafts space on a large table and began designing a lovely family album with all the photos we developed from our last family vacation. This took us three whole days and we enjoyed working together on this project* (31-y-old married woman, two children).

An important element in adjusting to this new situation is coping together as a couple. Members of a couple who are accustomed to working outside the home and seeing each other only several hours a day found themselves together for many hours. If one partner is not working, they may feel unneeded and useless. Such situations can lead to frustration and conflict between partners and within the family. The family as a unit must adjust to the new reality.

*It wasn't easy for me to see my husband at home, not working, depressed, incapable of doing the simplest everyday tasks. Unlike him, I gathered all my strength and tried to keep things running smoothly at home and not disrupt the normal routine* (43-y-old married woman, four children).

The research participants also included young single students, nine of whom were forced by the situation to return to their parents’ homes. These participants described how difficult this was, citing their feelings of having regressed by returning to live with their parents and siblings, a sense of having been robbed of their freedom. Yet the situation also provided an opportunity to renew their acquaintance and draw closer to their families.

*Over the last two days the situation began to get worse. The entire family at home all the time is challenging and really difficult. We are all at different ages and stages, and each of us wants to do what we feel like doing. It's especially tough for me because before COVID-19 I was usually not at home. I was studying or at work or out with my girlfriends and suddenly I can't even leave the house. It drives me crazy* (23-y-old single woman).

### Theme 3–‘It's hard to fall asleep and even harder to get up in the morning’: fears and concerns for self and family

All the research participants described their concerns about their own health and the health of their loved ones. Ten mentioned that people close to them were under quarantine or had tested positive for COVID-19, increasing their stress and sense of helplessness. They described being overwhelmed by worry due to the uncertainty about when it would end and what would happen to them and their loved ones.

*There are moments of crisis. Especially at night. Sometimes I feel I'm on the brink of losing hope. I have trouble coping with the ambiguity and uncertainty about how long this crisis will last. There's no definitive time limit, and I wonder how long I'll be able to be strong. How will things be when this is all over? What about the children? How long can they hang on? What about my parents? When I finish my daily routine I am flooded with worries and have trouble falling asleep* (43-y-old divorced woman, four children).

Thirty-three of the participants expressed concerns about older family members or those at risk due to pre-existing medical conditions. They worried about their parents and grandparents. These worries led to a sense of uncertainty about the future, anxiety and lack of control over the situation.

*My grandmother is in a high-risk group. She must not leave the house, and the grandchildren, including me, cannot visit her. At first I found it very difficult to discuss the situation with her. She continued going to the grocery store and following her regular routine. When all her activities closed down she understood that the situation is really problematic and dangerous for her. I try to speak to her as frequently as possible by phone and video calls with the other grandchildren. The situation is very worrisome and makes me sad, both the physical risk of the virus and the emotional toll, because she now feels more lonely than ever before. Not only will she feel alone, she'll also feel she's getting old, something she has always feared* (24-y-old single woman).

Some of the participants described how their concerns and fears affected their behaviour and emotions. They mentioned sleep disturbances, restlessness, irritability and difficulties in performing tasks. They felt depressed, anxious, sad and lonely as well as nervous, uncertain and helpless (Table 5).

*During this period I have practically stopped functioning. I constantly worry about what the situation will be in a few more days and where I'll be and how I should behave during this period. Since the outbreak of the virus, every day I am more worried and concerned about my family, my friends, the entire world. I'm always anxious and am not functioning like I used to. I'm more irritable and I don't feel like talking to anyone. I'm a student and I can't concentrate on my studies or work from home. I am not functioning* (28-y-old single woman).

## Discussion

The objective of the current study was to use a mixed-methods approach to examine psychological responses to the COVID-19 outbreak among the Israeli public. The findings indicate that about 48% of the public had negative emotional reactions and 20% believed they were likely to be infected with the virus. Moreover, a positive association was found between emotional responses and extent of the perceived threat. Emotional reactions were higher among younger participants, women and participants with higher perceived susceptibility. The qualitative study expanded our understanding of the psychological process people underwent. Participants described their sense of shock and chaos at the outbreak of the epidemic, followed by a gradual process of adjustment to the new situation along with fears and concerns for their own welfare and that of their loved ones.

The findings point to the presence of some degree of psychological distress among half the respondents, which, if not properly managed, has the potential to progress. The emergence of mental health issues in the wake of life-threatening events has been demonstrated among survivors of the Ebola and SARS outbreaks, who exhibited stress, worry and post-traumatic stress disorder (PTSD) symptoms.^23,24^ A large Italian study of 18 147 individuals during the COVID-19 lockdown found PTSD symptoms and depression among 37% of respondents.^25^ Examinations of the mental health status of the general population during the COVID-19 pandemic revealed signs of stress, anxiety and depression in Japan,^26^ Iran^27^ and Germany.^28^

Since COVID-19 is a global threat, all the news and media channels provide continuous coverage of the epidemic, and specifically of the Ministry of Health's updated statistics and most recent guidelines.^12^ The qualitative study revealed people's prevailing sense of confusion and chaos at the time of the outbreak, their need to readjust to their new situations and their concerns for their families. Moreover, most people locked down in their homes watch the news non-stop and tend to panic about the rising numbers of infections and deaths.^17,29^ Indeed, media exposure is another possible explanation for the high levels of stress and emotional response emerging in this study. The study was conducted about 3 mo after the initial COVID-19 outbreak and 1 mo after the crisis hit Israel. The Israeli public had already received health guidelines and information about the virus via the media. One Israeli study found that >80% ascribed the public's concerns over COVID-19 to media coverage of the outbreak.^30^ A study on COVID-19 conducted in India found that media exposure increased the public's anxiety level.^31^ Evidence indicates that repeated engagement with trauma-related media content for several hours a day shortly after a collective trauma may prolong acute stress.^32^ In the specific case of the COVID-19 outbreak, greater exposure to threat increases people's fears regarding the virus.^33^ Yet we must also bear in mind that such exposure can become quickly habituated.^34^

In the qualitative study, the participants’ emotional responses and sense of threat stemmed from their concerns for their own health and that of their loved ones, their worries about employment, their difficulties in staying home with their children and spouses and the problems posed by working at home. These findings are in line with findings of previous studies conducted during the COVID-19 outbreak pointing to a variety of concerns among research participants: health anxiety, personal health, the threat to loved ones, risk control, employment, virus spread and economic and societal consequences.^35,36^ Likewise, societal safety measures (e.g. lockdowns) have their use in preventing the spread of infections. Yet when such safety measures are too prolonged or too strict, they can have negative consequences, among them economic disruption and unemployment.^37^ Social distancing that includes closing shops and schools and working from home is likely to make people feel safer but also to increase their feelings of isolation, stress and frustration and to cause difficulties in many life situations (in the family, between members of a couple, in the sphere of employment).^38^ Another major source of fear regarding COVID-19 was the perceived risk of loved ones being infected. This fear can be mitigated by providing the general public with clear information about the risks and by taking (additional) steps to protect vulnerable groups at risk of infection.^3,4^

This study has several limitations. Most of the study participants were female. In addition, the qualitative research questions sought to understand the research participants’ feelings, behaviours and means of coping with the coronavirus crisis. Nevertheless, they limited the possibilities for deriving additional authentic information from the participants, such as how they coped with economic issues and changes in spousal relations. Given that the survey was conducted during the fourth week of the virus outbreak in Israel, it portrays an immediate and initial picture of the reactions of the general public to COVID-19. As the virus continues to spread, the behavioural guidelines are constantly being changed in light of the rising morbidity and mortality rates in Israel and worldwide. Therefore, research should continue to explore psychological and emotional responses among the general public over time. The question regarding the resources for coping with COVID-19 was a single choice question rather than giving respondents the possibility of different options. The validity of answers is a general problem of online surveys, which we attempted to address by the differential approach described in the Methods section. The use of a mixed-methods approach limits the ability to generalise our results to a wider population and to make claims about directionality. Therefore, conclusions about directionality or causality in the relationships should be treated with caution.

## Data Availability

The authors have the research data, which are available upon request.

## Appendix 1: Appendix 1: Interview questions posed to study participants

1. How do you feel about the coronavirus?
2. Tell me about your behaviour since the outbreak of the virus.
3. How has the outbreak of the virus affected you?
4. How has the outbreak of the virus affected your feelings about and the way you conduct yourself with people close to you (friends/relatives)?
5. Tell me about your thoughts and feelings about the present and the future.
6. What can help you during this period?
